# Analytical data on respiratory symptoms and pulmonary impairments due to exposure to non-combusted liquefied petroleum gas

**DOI:** 10.1016/j.dib.2021.107106

**Published:** 2021-04-29

**Authors:** Emmanuel Akpan, Imoh Moffat, Ukeme Abara

**Affiliations:** a Federal School of Medical Laboratory Technology (Science), Jos, Nigeria; b Department of Mathematical Sciences, Abubakar Tafawa Balewa University, Bauchi, Nigeria; c Department of Statistics, University of Uyo, Uyo, Nigeria; d Department of Medical Laboratory Science, University of Calabar, Nigeria

**Keywords:** Environmental pollution, Liquefied petroleum gas, Occupation health, Public Health, Pulmonary Function, Respiratory Symptoms

## Abstract

The article is aimed at assessing the effects of chronic exposure to non-combusted LPG on the prevalence of respiratory symptoms and appraising the potential pulmonary impairments among LPG vendors. A case control design in which vendors and non-vendors of LPG from Calabar, Nigeria were used for the data collection. Seventy five (75) apparently healthy LPG vendors and Seventy five (75) apparently healthy non LPG vendors, aged 18 to 50 years were considered. A structured questionnaire was randomly administered to the participants to obtain information on age, family history, medical history, physical lifestyle, drug usage, occupation and duration on the job. The Forced expiratory volume in 1 second (FEV_1_), forced vital capacity (FVC) and peak expiratory flow (PEF) were obtained using a Spirometer while FEV_1_/FVC was calculated. Independent *t*-test was applied to determine the mean difference between the exposed and control groups at 5% level of significance. Chi-square test/Fisher's exact test was used to investigate all forms of associations in the study. It is evident in the data that nasal irritation/sneezing and cough were significantly associated with the LPG vendors. The pulmonary function parameters except FEV_1_/FVC indicated significant reduction among LPG vendors. The data can further be reused by applying regression analysis, correlation analysis to determine the relationship between pulmonary function indices and duration of exposure. Also, analysis of variance (ANOVA) can be used for determining the effect of interaction between age of exposed group and duration of exposure on pulmonary function parameters.

## Specifications Table

**Table:** 

Subject	Public Health and Health Policy
Specific subject area	Environmental and Occupational Health, Biostatistics
Type of data	Table Text file
How data were acquired	Primary data based on structured questionnaire and the pulmonary function indices of the participants were measured with a portable office SP10 Spirometer
Data format	Raw Calculated Partially Analyzed Tabulated An excel file with data has been uploaded
Parameters for data collection	Vendors and non-vendors of Liquefied Petroleum Gas (LPG)
Description of data collection	A case control design in which vendors and non-vendors of LPG were used for the data collection. Seventy five (75) apparently healthy LPG vendors and Seventy five (75) apparently healthy non LPG vendors, aged 18 to 50 years were considered. The data were collected using a structured questionnaire and Spirometer. The questionnaire is provided as a supplementary file.
Data source location	Calabar, Cross River State, Nigeria
Data accessibility	Data included in this article. Repository name: Mendeley Data Data identification number: 10.17632/7fgpr9tk8h.1 Direct URL to data: http://dx.doi.org/10.17632/7fgpr9tk8h.1

## Value of the Data

• The data is useful for assessment of respiratory symptoms and pulmonary function for the purpose of investigating and monitoring of LPG vendors with and at risk of respiratory abnormalities.
• The evidence provided by the data could help in designing and implementing policy to protect and promote health of LPG vendors.
• The data can further be analyzed using other statistical tools such as regression analysis, correlation analysis (for determining the relationship between pulmonary function indices and duration of exposure) and ANOVA Analysis (for determining the effect of interaction between age of exposed group and duration of exposure on pulmonary function indices).

## 1 Data Description

Acute inhalation of liquefied petroleum gas (LPG) has been associated with death through respiratory system attacks and associated respiratory symptoms often identified are wheeze, cough, chest tightness, nasal irritation/sneezing, dizziness and drowsiness [1,2]. There are guidelines that checkmate the level of exposure to harmful substances such as LPG. For example, Acute Exposure Guideline Levels (AEGLs) for hazardous substances are well documented in [3].

In addition, different recommendations for occupational safety and health have been made and are meant to protect workers’ health and safety over a working lifetime. According to [2], such recommendations are called Recommended Exposed Limits (RELs). REL is frequently expressed as a time-weighted average (TWA) exposure for up to 10 hr/day during a 40-hr work week. In term of a short-term exposure limit, the exposure time of 15 min should never be exceeded while the ceiling limit that should never be exceeded even instantaneously unless specified over a time period and shall be assessed as a 15 min TWA exposure.

The works of [4], [5], [6], [7] showed a rise in the systolic blood pressure (SBP) and diastolic blood pressure (DBP) due to exposure to LPG. Meanwhile [8,9], provided evidence of high prevalence of health-related symptoms among those exposed to LPG. Moreover, different studies have shown evidence of negative effects of health conditions including impaired pulmonary function as a result of indoor exposure to cooking gas [7,^8^,[10], [11], [12], [13]. In this article, a chronic exposure to LPG is considered and the dataset used can be assessed as supplementary data and the questionnaire used is provided as a supplementary file. The data can be analyzed using independent *t*-test, Chi-square test, analysis of variance, regression analysis and correlation analysis.

The description of the characteristics of the participants is given in Table 1. Hundred and fifty participants took part in the study, 75 were cooking gas vendors exposed to LPG and one out of the 75 participants has a life style of drinking alcohol while 75 non vendors not exposed to LPG. The SBP (122.97 mmHg), DBP (81.06 mmHg) and BMI (26.02 kg/ m^2^) for the gas vendors are higher compared to the SBP (119.53 mmHg), DBP (78.89 mmHg) and BMI (21.24 kg/ m^2^) for the non vendors with significant difference (*p* < 0.05) in these subjects.

**Table 1:** Description of study participants.

	Vendor			Non Vendor				
Characteristics	N	Mean	SD	N	Mean	SD	*T*-value	*P*-value
SBP(mmHg)	75	122.97	2.66	75	119.53	1.36	9.975	< 0.001⁎⁎
DBP(mmHg)	75	81.06	3.19	75	78.89	1.32	5.455	< 0.001⁎⁎
BMI (kg/m^2^)	75	26.02	4.85	75	21.24	3.06	7.216	< 0.001⁎⁎

*Abbreviations:* LPG = liquefied petroleum gas, SD = standard deviation, SBP = systolic blood pressure, DBP = diastolic blood pressure, BMI = body mass index.

*Significant at 0.05 level.

⁎⁎ Significant at 0.01 level.

Among the respiratory symptoms considered in LPG vendors, nasal irritation/sneezing has the highest rate of prevalence (56%), followed by cough (53.3%), wheeze (40%) and chest tightness (26.7%), respectively (Table 2).

**Table 2:** Prevalence of respiratory symptoms in LPG vendors.

Respiratory Symptoms	*N*	Frequency	Prevalence (%)
Wheeze	75	30	40
Cough	75	40	53.3
Chest tightness	75	20	26.7
Nasal irritation/Sneezing	75	42	56

The prevalence of nasal irritation/sneezing and cough was found to be significantly associated with the LPG vendors given that the hypothesis of no association was rejected with probability value related to the Chi square ($\chi^{2}$) statistic is 0.001, respectively and is less than 5% significance level. Otherwise, the prevalence of wheeze and chest tightness appeared to show no significant association with the corresponding probability value being greater than 5% significance level (Table 3).

**Table 3:** Association between respiratory symptoms and LPG vendors.

											Nasal		
		Wheeze			Cough			Chest tightness			Irritation/Sneezing		
		Yes	No	Total	Yes	No	Total	Yes	No	Total	Yes	No	Total
Vendor	Nonseller	25	50	75	20	55	75	13	62	75	21	54	75
	Seller	30	45	75	40	35	75	20	55	75	42	33	75
	Total	55	95	150	60	90	150	33	117	150	63	87	150
	$\chi^{2}$	0.718			11.111			1.904			12.069		
	*P*-value	0.094			0.001⁎⁎			0.237			0.001⁎⁎		

*Significant at 0.05 level.

⁎⁎ Significant at 0.01 level.

The respiratory symptoms were found to be independent of age at *P* > 0.05 as shown in Table 4. The hypothesis of no association between respiratory symptoms and different age groups was not rejected. For chest tightness and age, Fisher's exact test was used since more than 20% of cells have expected frequencies less than 5. The Fisher's exact test value is 1.268 with the corresponding p-value of 0.781 greater 5% significance level. The Chi-square ($\chi^{2}$) values for wheeze, cough and nasal irritation/sneezing against age are 1.147, 1.983 and 0.117 with the corresponding p-values of 0.776, 0.596 and 1.000 which are greater than 5% significance level.

**Table 4:** Association between respiratory symptoms and age of LPG vendors.

											Nasal		
		Wheeze			Cough			Chest tightness			Irritation/Sneezing		
		Yes	No	Total	Yes	No	Total	Yes	No	Total	Yes	No	Total
	18–27 years	9	12	21	11	14	25	5	17	22	12	9	21
Age	28–37 years	6	14	20	9	8	17	5	16	21	10	9	19
	38–47 years	8	10	18	10	5	15	5	14	19	12	9	21
	48 years and Above	7	9	16	10	8	18	5	8	13	8	6	14
	Total	30	45	75	40	35	75	20	55	75	42	33	75
	$\chi^{2}/$**Fisher's** Exact Test	1.147			1.983			**1.268**			0.117		
	*P*-value	0.776			0.596			**0.781**			1.000		

There was no association between respiratory symptoms and duration of exposure at (*P* > 0.05) as shown in Table 5. The hypothesis of no association between respiratory symptoms and duration of exposure was not rejected given that the Chi-square ($\chi^{2}$) values for wheeze, cough, chest tightness and nasal irritation/sneezing against duration of exposure are 3.606, 4.075, 0.663 and 1.347 with corresponding p-values of 0.176, 0.130, 0.758 and 0.515 which are greater than 5% significance level.

**Table 5:** Association between respiratory symptoms and duration of exposure.

											Nasal		
		Wheeze			Cough			Chest tightness			Irritation/Sneezing		
		Yes	No	Total	Yes	No	Total	Yes	No	Total	Yes	No	Total
	1–3years	10	19	29	13	17	30	7	22	29	15	14	29
Duration of Exposure	4–6 years	8	17	25	15	6	21	6	19	25	13	12	25
	Above 6 years	12	9	21	12	12	24	7	14	21	14	7	21
	Total	30	45	75	40	24	75	20	55	75	42	33	75
	$\chi^{2}$	3.606			4.075			0.663			1.347		
	P-value	0.176			0.130			0.758			0.515		

The lung function test as indicated in Table 6 showed that the means of FEV_1_, FVC and PEF were higher in the non-vendor group (FEV1: 3.594±0.702; FVC: 3.789±0.679; FEV_1_/FVC: 0.390±0.971; PEF: 7.117±1.397) except FEV_1_/FVC as compared with the vendor group (FEV_1_: 1.925±0.637; FVC: 2.037±0.738; FEV_1_/FVC: 1.009±0.124; PEF: 4.151±1.258). The assessment showed a significant decrease in the pulmonary function indices (FEV_1_, FVC and PEF) of the vendors with respective p-value of 0.001 which is less than 5% significance level with exception to FEV_1_/FVC whose p-value is 0466.

**Table 6:** Pulmonary function assessment.

	Vendor			Non Vendor				
Parameters	N	Mean	SD	N	Mean	SD	T-value	P-value
FEV1 (liters)	75	1.925	0.637	75	3.594	0.702	−15.255	< 0.001^⁎⁎^
FVC (liters)	75	2.037	0.738	75	3.789	0.679	−15.136	< 0.001^⁎⁎^
FEV1/FVC	75	1.009	0.124	75	0.390	0.971	0.229	0.466
PEF	75	4.151	1.258	75	7.117	1.397	−13.665	< 0.001^⁎⁎^

*Abbreviation:* FEV1 = forced expiratory volume in one second, FVC = forced vital capacity, FEV1/FVC = FEV1, FVC ratio, PEF = peak expiratory flow, SD = standard deviation.

## 2 Experimental Design, Materials and Methods

A case control design in which vendors and non-vendors of LPG were used for the study. The sizes of cylinders refilled ranged from 4 kg to 50 kg. The amount sold per participant each day was obtained from their record books over the period of two weeks. The average of this was 755 kg and was taken to indirectly represent the daily LPG exposure since we were not able to directly determine the amount of LPG escaping into the ambient air, hence the actual amount in ppm (parts per million) exposed to by each participant could not be ascertained and as such, the measurement of the actual amount (in ppm) exposed to by the subjects need further research. A total of 150 subjects were recruited which consist of seventy five (75) apparently healthy LPG (cooking gas) vendors and seventy five (75) apparently healthy non gas vendors /users (control). The inclusion criteria for the exposed group were: residence in Calabar, Cross River State, Nigeria; age ranging from 18 to 50 years, having at least one year exposure to LPG and selling for at least 6 h daily, and devoid of respiratory diseases history before commencing the trade. The inclusion criteria for the control were: residence in Calabar, age ranging from 18 to 50 years, apparently healthy, no work-related exposure to LPG and devoid of history of hospitalization due to respiratory diseases.

A structured questionnaire was randomly administered to the participants to obtain information on age, family history, medical history, physical lifestyle, drug usage, occupation and duration on the job. Cooking gas vendors who consistently sold it at least for the past one year as at the time of this study and non LPG vendors /users who never sold or used gas (control) were recruited into the study.

The lung function of the participants was measured with a portable office SP10 Spirometer. It was calibrated following approved procedures [14]. The following Lung function parameters were measured; forced expiratory volume in 1 second (FEV_1_), forced vital capacity (FVC) and peak expiratory flow (PEF). FEV_1_/ FVC ratios were mathematically calculated [15], [16], [17].

## Ethics Statement

Ethical approval was sought and obtained from the Health Research and Ethics committee of the Cross River State Ministry of Health, Nigeria. The purpose and nature of the research was explained to the participants and written consent was obtained.

## Declaration of Competing Interest

The authors declare that they have no known competing financial interests or personal relationships which have, or could be perceived to have, influenced the work reported in this article.

## Appendix Supplementary materials

Image, application 1
