# Supplementary material for: Analytical data on respiratory symptoms and pulmonary impairments due to exposure to non-combusted liquefied petroleum gas
Source: Data Brief. 2021 Apr 29;36:107106. doi: 10.1016/j.dib.2021.107106 (PMC8257966; doi:10.1016/j.dib.2021.107106)
Supplement: Supplementary file 1 [file mmc1.docx]

**A Questionnaire on Respiratory Symptoms and Pulmonary Impairments due to Exposure to Non-combusted Liquefied Petroleum Gas (LPG) among Sellers in Calabar**

**Target Respondents:** To be completed by selected sellers and non-sellers of LPG in Calabar.

**Aim:** The questionnaire is intended to collect data about the effects of chronic exposure to non-combusted LPG on the prevalence of respiratory symptoms and the potential pulmonary impairments among LPG vendors.

**Confidentiality:** Please note that your responses will be treated as anonymous and confidential.

Participant’s ID ______

**PART ONE: BIODATA OF PARTICIPANTS**

1. Age (years) : ________
2. Sex: male [ ] Female [ ]
3. Level of education: No formal education [ ]Primary [ ]secondary [ ] Tertiary [ ]
4. Marital Status: Single [ ] Married [ ]
5. Occupation: Gas seller [ ] others………………………………

(Please specify)

**PART TWO: DURATION OF EXPOSURE**

1. Duration on the Job: 1-3 years [ ], 4-6 years [ ], Above 6 years [ ]

(Please specify)

1. Number of hours worked daily: 6 hours [ ], 7-8hours [ ], 9 hours and above [ ]

**PART THREE: ANTHROPOMETRIC MEASUREMENT**

1. Weight (kg) ………, height (meters) ………., BMI (kg/m²) ………….
2. Blood pressure: systolic (mmHg) ……., diastolic (mmHg) …………...

**PART FOUR: LIFESTYE INFORMATION**

1. Do you smoke? Yes [ ], No [ ]
2. Do you drink alcohol? Yes [ ], No [ ]

**PART FIVE: HEALTH INFORMATION**

1. Have you ever been diagnosed of any of diabetes, hypertension, heart

disease, kidney disease or lung disease? Yes [ ], No [ ]

1. Are you currently on any medication? Yes [ ], No [ ]
2. Have you ever been diagnosed of any respiratory tract diseases?

Yes [ ], No [ ]

**PART SIX: EXPOSURE TO LPG**

1. Do you frequently experience wheezing or noise in your chest since you started your occupation?

Yes [ ], No [ ]

1. Do you have persistent cough (cough for more than 3 consecutive weeks)?

Yes [ ], No [ ]

1. Do you experience difficulty breathing or feeling of chest tightness

breathing? Yes [ ], No [ ]

1. Do you experience nasal irritation/sneezing? Yes [ ], No [ ]
2. Do you perceive the smell of gas breath long after selling gas?

Yes [ ] , No [ ] (for gas sellers only)

1. Do you wear personal protective selling gas? Yes [ ], No [ ]
2. Do you use gas for cooking? Yes [ ], No[ ] (for non gas sellers only)

**PART SEVEN: VALUES OF LUNG FUNCTION INDICES AS MEASURED BY**

**SPIROMETER**

1. Forced expiratory volume in one second (FEV1) ……………
2. Forced vital capacity (FVC) ……………
3. FEV1/FVC ……………
4. Peak expiratory flow (PEF) ……………
